# Cinnamon extract inhibits angiogenesis in zebrafish and human endothelial cells by suppressing VEGFR1, VEGFR2, and PKC-mediated MAP kinase

**DOI:** 10.1002/fsn3.13

**Published:** 2013-01-08

**Authors:** Rishipal R Bansode, TinChung Leung, Priscilla Randolph, Leonard L Williams, Mohamed Ahmedna

**Affiliations:** 1 Center for Excellence in Post Harvest Technologies, North Carolina Agricultural and Technical State UniversityNorth Carolina Research Campus, Kannapolis, North Carolina, 28081; 2 Biomedical/Biotechnology Research Institute, Department of Biology, North Carolina Central University, Nutrition Research Center, North Carolina Research CampusKannapolis, North Carolina, 28081; 3 Department of Health Sciences, Qatar UniversityP.O. Box 2713, Doha, Qatar

**Keywords:** Angiogenesis, cinnamon, mitogen-activated, protein kinase, protein kinase C, vascular endothelial growth factor receptor

## Abstract

Angiogenesis is a process of new blood vessel generation and under pathological conditions, lead to tumor development, progression, and metastasis. Many bioactive components have been studied for its antiangiogenic properties as a preventive strategy against tumor development. This study is focused on the effects of cinnamon extract in modulating the pathway involved in angiogenesis. Human umbilical vein endothelial cells (HUVEC) were treated with cinnamon extract at a concentration of 25 μg/mL for 1, 3, or 6 h followed by treatment with phorbol ester (TPA) at a concentration of 10 nmol/L to induce mitogen-activated protein kinase (MAPK) expression. Results show that cinnamon extract inhibited TPA-induced phosphorylation of MAPK and AKT in a dose-dependent manner. Gene expression results in HUVEC showed that cinnamon extract treatment inhibited TPA induction of protein kinase C, PKCα and PKCη messenger RNA (mRNA) expression in a dose-dependent manner along with suppression of vascular endothelial growth factor receptor 1 (VEGFR1/Flt1) and vascular endothelial growth factor receptor 2 (VEGFR2/KDR/Flk1) mRNA expression. Cinnamon extract was administered to zebrafish embryos during gastrulation at 6–8 h post fertilization (hpf). The embryos were observed for changes in morphology, toxicity, and blood vessel development. The intersegmental vessels in the zebrafish embryos were attenuated and underdeveloped at an effective cinnamon extract dose of 250 μg/mL compared with the DMSO-treated control. Exposure to cinnamon extract for 36 h resulted in gross morphological deformities. The results suggest the effect of cinnamon extract on angiogenesis is mediated by PKC-dependent phosphorylation of MAPK.

## Introduction

Medicinal properties of herbal plants have been extensively investigated and have become the focus of alternative medicine in treating different diseases, including cancer, diabetes, and obesity (Kwon et al. 2010). *Cinnamomum cassia* bark belongs to the family of Lauraceae, which contains large amounts of bioactive molecules, including essential oils (cinnamic aldehyde and cinnamyl aldehyde), tannin, mucus, and carbohydrates (Kwon et al. 2009).

The bioactive properties of cinnamon have been studied in a wide range of biological functions, including anti-inflammatory (Lee et al. 2005), antioxidant (Shan et al. 2009), antimicrobial (Matan et al. 2006; Shan et al. 2009), and antidiabetic effects (Khan et al. 2003). In addition to its effects on angiogenesis, metastasis, and cell survival (Jochum et al. 2001; Garg and Aggarwal 2002; Karin et al. 2002), cinnamon extract has been demonstrated by Kwon et al. (2010) to act as an antimelanoma agent by targeting angiogenesis and the cytolytic effector function of CD8^+^ T cells.

Vascular endothelial growth factor (VEGF) is a potent angiogenic factor that is induced by hypoxia and plays a central role in the development of neovascularization in multiple diseases, including tumor growth (Suzuma et al. 2002). In recent times, research has focused on creating therapies that could interfere with angiogenesis by targeting VEGF proteins. VEGF can activate several protein kinase C (PKC) isoforms, including α, β1, and β2, and δ isoforms (Xia et al. 1996; Aiello et al. 1997). One of the mechanism with which it exerts angiogenesis is through mitogen-activated protein kinase (MAPK)-mediated activation of the extracellular signal-regulated kinase (ERK; Niimi et al. 2001). Extracellular signal-regulated kinases ERK1 and ERK2 are phosphorylated in response to angiogenic stimuli-mediated by VEGF (Zachary 2003) and basic fibroblast growth factor (Pintucci et al. 2002), as wells as phorbol ester (TPA; Kuzuya et al. 1999).

Tetradecanonoylphorbol-13-acetate (TPA) is a diester of phorbol and a potent tumor promoter that is often employed in biomedical research to activate the signal transduction enzyme PKC (Castagna et al. 1982; Niedel et al. 1982; Blumberg 1988). The effects of TPA on PKC result from its similarity to one of the natural activators of classic PKC isoforms, diacylglycerol. Activation of PKC by TPA has been shown to affect cultured endothelial cells, including induction of migration, proliferation, and vessel formation (Montesano and Orci 1985; Taylor et al. 2006).

Although antiangiogenic properties of cinnamon has been established (Kwon et al. 2009; Lu et al. 2010) a precise mechanism with which cinnamon extract exerts antiangiogenic effect has remained elusive. We hypothesized that cinnamon exerted antiangiogenic property by inhibiting PKC-dependent MAPK regulation of VEGF receptors. In order to test this hypothesis, we evaluated the key angiogenic effectors' response to cinnamon extract *in vitro* using endothelial cells (HUVEC) by inducing phosphorylation of MAPK in the presence of TPA, a known activator of PKC enzymes. The study also sought to demonstrate that cinnamon exhibits antiangiogenic activity when tested in a zebrafish model system *in vivo*.

## Material and Methods

### Cinnamon-extract preparation and properties

One hundred milligrams of dry cinnamon (*C. cassia*) bark powder (McCromick & Co., Inc., Hunt Valley, MD) was dissolved in DMSO and agitated overnight at 4°C as described by Cao and Anderson (2011). The sample was then centrifuged at 2000 g for 10 min at 5°C and the supernatant was separated and filtered with 0.2 μ syringe filter. The extract was stored at −20°C until use for cell culture assays and zebrafish studies.

### Cell Viability assay

Human umbilical vein endothelial cell line, HUVEC, were obtained from Lonza (Lonza, Wakersville, MD) and were grown in minimum complete endothelial growth media supplemented with 2% fetal bovine serum, bovine brain extract, hEGF, hydrocortisone, ascorbic acid, gentamycin, and amphotericin B (Lonza). The cells were cultured at 37°C in a humidified atmosphere of 95% air-5% CO_2_.

Once the monolayer had become approximately 80% confluent, the cells were seeded, at a density of 5000 cells per well, in a 96-well plate. The cells were later treated with cinnamon extract at doses of 0–200 μg/mL for a period of 24 h. Before the end of the experiment, 50 μL of XTT labeling mixture (125 μmol/L 2,3-bis (2-methoxy-4-nitro-5-sulfophenyl)-5-[(phenylamino) carbonyl]-2H-tetrazolium hydroxide (XTT) and 25 μmol/L *N*-methyl dibenzopyrazine methyl sulfate [PMS]) per well was added and plate was incubated for further 4 h at 37°C and 5% CO_2_. The spectrophotometric absorbance of the formazan product was measured using Synergy 2 multimode microplate reader (BioTek, Winooski, VT). The wavelength to measure absorbance of the formazan product was 475 nm, and the reference was 650 nm.

### RT-PCR study for gene expression

HUVECs were treated with cinnamon extract at a dosage of 10 and 25 μg/mL for 3 h in the presence of TPA at a concentration of 10 nmol/L for 2 h. Total RNA was isolated from HUVECs after 3 h of incubation with cinnamon extract. The amplification was performed using Eppendorf realplex-RT-PCR (Eppendorf, Hauppauge, NY) using the following set of primers: PKCα sense: 5′-TGGCAAAGGAGCAGAGAACT-3′, antisense: 5′-TGTAAGATGGGGTGCACAAA-3′; PKCη sense: 5′-AGTAGACTGGTGGGCAATGG-3′, antisense: 5′-GATCCCTGTGGCATCTTCAT-3′ VEGFR1: sense: 5′-TTTGGATGAGCAGTGTGAGC-3′, antisense: 5′-CGGCACGTAGGTGATTTCTT-3′; VEGFR2: sense: 5′-AGCGATGGCCTCTTCTGTAA-3′, antisense: 5′-ACACGACTCCATGTTGGTCA-3′; β-actin: sense: 5′-CTCTTCCAGCCTTCCTTCCT-3′, and antisense: 5′-AGCACTGTGTTGGCGTACAG-3′. The PCR amplification was performed using Eppendorf's Masterplex® realplex thermal cycler (Eppendorf) under the following conditions: 30 cycles at 94°C for 1 min, 56°C for 1 min, and 72°C for 2 min followed by 10 min at 72°C. The final product was subjected to electrophoresis on a 12% polyacrylamide gel and detected by ethidium bromide staining via a UV light using a CCD camera (Fotodyne Inc., Hartland, WI). The relative expression levels of the mRNAs of the target genes were normalized using the β-actin internal standard using ImageJ software (NIH, Bethesda, MD).

### Western blot assay for MAPK and AKT protein expression

Human umbilical vein endothelial cells were treated with 25 μg/mL cinnamon extract for 1, 3, or 6 h followed by treatment with phorbol ester (TPA) at a concentration of 10 nmol/L for 5 minutes to induce MAPK expression as described by Zavoico et al. (1990). A negative control consisting of HUVEC cells exposed to highly selective inhibitor of both MAPK kinases MEK1 and MEK2 (U0126, Sigma-Aldrich, Co., St. Louis, MO) was incubated for 30 min followed by addition of TPA for 5 min. The positive control consisted of treatment of HUVEC cells with TPA alone while the control sample involved treatment with DMSO. The cells were harvested using Laemmli buffer containing 5 mmol/L DTT. The cell lysates were electrophoresed on a polyacrylamide gel then transferred onto nitrocellulose membrane. Immunoblotting was performed on the nitrocellulose membrane using phospho-specific primary antibody for MAPK^42/44^ (Cell Signaling, Danvers, MA) and AKT Thr^308^ (Cell Signaling), and nonphospho-specific MAPK^42/44^ (Santa Cruz Biotechnology Inc., Santa Cruz, CA) and AKT (Cell Signaling). As the secondary antibody, horseradish peroxidase-conjugated anti-rabbit, or anti-mouse antibody (Cell Signaling) were applied, respectively. Electrochemiluminescence reagent (Thermo Scientific, Rockford, IL) was used for detection and was visualized using CCD camera (Fotodyne Inc.). The relative expression levels of the phosphorylated MAPK and AKT were normalized using the total protein expression levels of MAPK and AKT, respectively, using ImageJ software (NIH).

### Zebrafish husbandry

Zebrafish embryos, obtained from natural spawning of *Tg*(*flk1:GFP*) transgenic line, expressing green fluorescent protein (GFP) under the vascular endothelial growth factor receptor 2 (*vegfr2/kdr/flk1*) promoter, were kindly provided by Dr. Suk-Won Jin (Yale Medical School, New Haven, CT) for use in this study. The zebrafish were maintained in multiphase filtration stand-alone zebrafish systems (Aquaneering, Inc., San Diego, CA) at 28.5°C on a 14/10-h (light/dark) photoperiod. The husbandry and treatment of zebrafish embryos was described previously by Leung et al. (2006). All experimental procedures using zebrafish research were approved by the Animal Ethical Committee of the North Carolina Central University IACUC Protocol # TCL-07-14-2008.

### Zebrafish morphology and toxicity assays

Cinnamon extracts were administered to zebrafish embryos at 6–8 h post fertilization (hpf) at a concentration of 0, 50, 100, 150, 200, 250, 500, and 1000 μg/mL in embryo medium (0.3× Danieau's solution containing 19.3 mmol/L NaCl, 0.23 mmol/L KCl, 0.13 mmol/L MgSO_4_, 0.2 mmol/L Ca(NO_3_)2, 1.7 mmol/L HEPES, pH 7.0). The duration of exposure was 16 and 36 h. Triplicates of 10 embryos were placed per well (*n* = 30).

The embryos with 16-h exposure were observed for morphological, toxicity and blood vessel development changes. The zebrafish embryos were then rescued and transferred in embryo medium containing 0.003% 1-phenyl-2-thiourea (PTU) medium to inhibit pigment formation and incubated for additional 24 h and imaged for blood vessel development at 48 hpf using an Olympus MVX10 MacroView Fluorescence Microscope (Olympus, Center Valley, PA) with Hamamatsu C9300-221 high-speed digital CCD camera (Hamamatsu City, Japan). *Tg*(*flk1:GFP*) transgenic zebrafish with fluorescent blood vessels was used to facilitate image analysis. In another experiment, zebrafish embryos were continuously exposed to cinnamon extract for a period of 40 h and imaged at 48 hpf. The embryos were observed for morphological phenotypes, heart rate, and intersegmental vessel (ISV) abnormalities. The ISV length and diameter of zebrafish embryos were assessed using NIH ImageJ software (NIH).

### Statistical analysis

A two-tailed Student's *t*-test or one-way ANOVA plus Newman–Keuls multiple comparison tests was employed to compare treatment means to the control where *P *<* *0.05 was considered to be statistically significant. Statistical analysis was conducted using SAS 9.2 software (SAS Inc., Cary, NC). Descriptive results of continuous variables were expressed as mean values ± SEM from three experiments.

## Results

### Cytotoxicity of cinnamon extract on HUVEC

**Figure 1:**
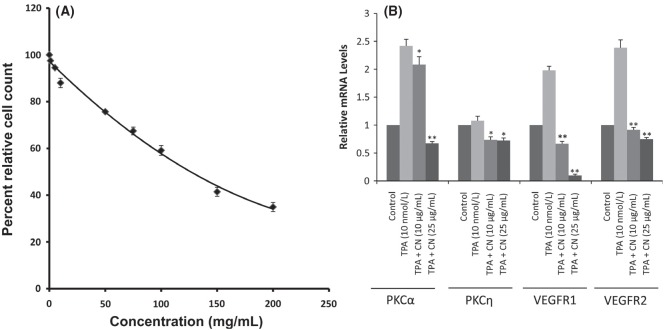
(A) Cytotoxicity of cinnamon extract on HUVEC cell line. Each treatment was run in triplicate. (B) RT-PCR gene expression results for HUVECs treated with doses of 10 and 25 μg/mL cinnamon extract in presence of 10 nmol/L TPA (positive control). HUVEC, human umbilical vein endothelial cells. *Significantly different from positive control at *P* < 0.05; **significantly different from positive control at *P* < 0.01.

Cinnamon extract exhibited toxicity toward HUVEC cells in a dose-dependent manner. Cinnamon significantly reduced the HUVEC cell viability at concentrations >50 μg/mL (Fig. 1A). Hence, subsequent experiments on HUVEC were performed after cinnamon extract treatment with concentrations in the range of 0–25 μg/mL.

### Inhibitory effects of cinnamon extract on TPA-induced PKC expression in HUVEC

In order to understand the cellular signaling pathway regulating the inhibition of the expression of the VEGF receptors in the presence of cinnamon extract, we investigated the role of PKC in regulating the angiogenic pathway. To test this hypothesis, an experiment was conducted to stimulate PKC activity in the presence of phorbol ester (TPA), a know activator of PKCs. The mRNA expression levels in TPA-treated cells showed increased expression of PKCα compared with the control. Cinnamon treatment in HUVECs treated with TPA significantly inhibited PKCα expression in a dose-dependent manner and partially suppressed PKCη levels as shown in Figure 1B. The mRNA levels of VEGFR1 and VEGFR2 increased under the influence of TPA treatment alone and reduced in presence of cinnamon treatment. The inhibition of VEGFR1 was greatly influenced by the exposure by cinnamon treatment followed by inhibition of VEGFR2 expression. The results strongly suggest that cinnamon exerts its angiogenic activity by modulating PKC activity, especially PKCα-mediated activation of VEGFR1 and VEGFR2.

### Effects of Cinnamon extract on TPA-induced phosphorylation of MAPK and AKT in HUVEC

**Figure 2:**
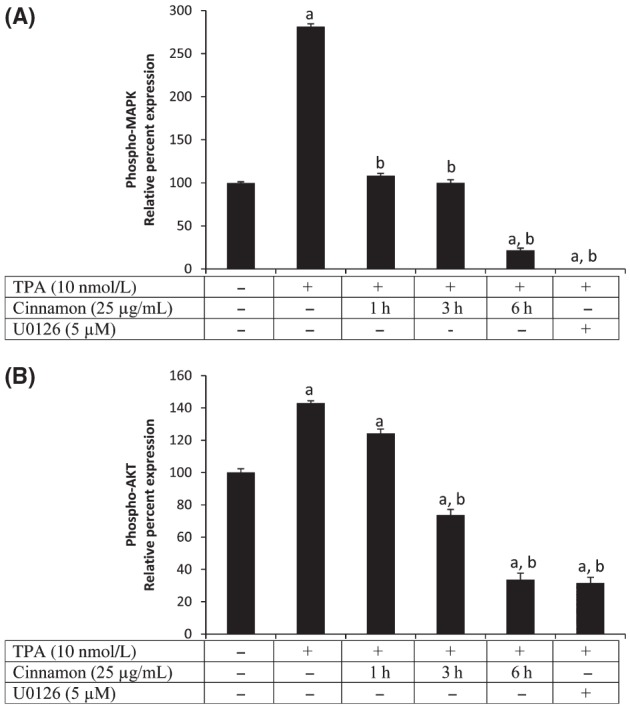
Western blot showing suppression of (A) phospho-mitogen-activated protein kinase and (B) phospho-AKT in presence of cinnamon extract exposed at a concentration of 25 μg/mL for 3 h in presence of 10 nmol/L TPA. ^a^Significantly different from control at *P* < 0.05; ^b^Significantly different from TPA-induced control at *P* < 0.05.

Phorbol ester is a well-known activator of PKCs as it mimics diacylglycerol by subsequently phosphorylating MAPK. Numerous investigators have demonstrated activation of MAPK^42/44^-phosphorylation by phorbol ester within 5 min of exposure (Braconi Quintaje et al. 1998; May et al. 1998; van Rossum 2001). We observed that cinnamon extract prevented TPA-induced MAPK^42/44^ activation by inhibiting TPA-induced phosphorylation of MAPK^42/44^, without effecting total MAPK^42/44^. Results show that cinnamon extract inhibited TPA-induced phosphorylation of MAPK in a dose-dependent manner (Fig. 2A). We also observed a significant decrease in phosphorylation of AKT upon exposure of cinnamon extract to the TPA-induced HUVEC. This effect was already apparent within 1 h of treatment and was significantly reduced between 3 and 6 h of treatment (Fig. 2B). The data also suggest that the inhibitory role of cinnamon extract against angiogenesis involves PKC stimulation. Taken together, these results demonstrate that inhibition of angiogenic activity by cinnamon is mediated by PKC signaling via VEGF receptor inhibition in a MAPK-dependent manner.

### Cinnamon extract affects zebrafish ISVs formation

**Figure 3:**
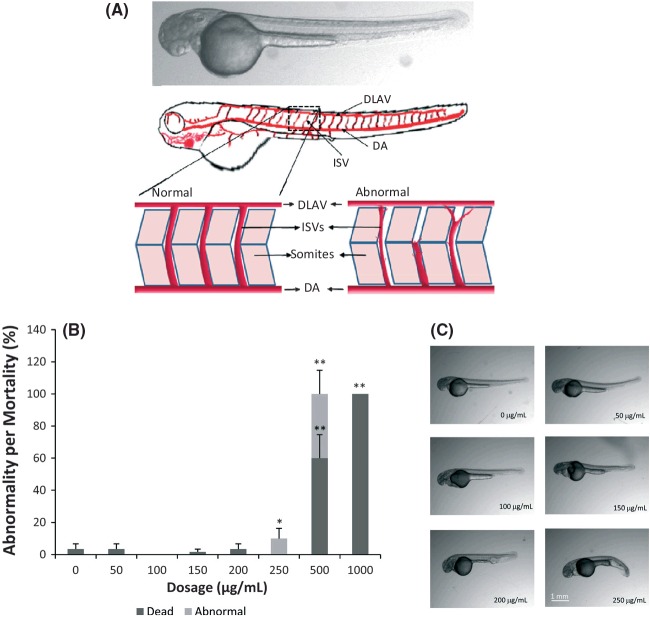
(A) Illustration of zebrafish highlighting the trunk region where intersegmental vessel (ISVs) develop between somites. The sprouting of ISVs results in an interconnected luminal pathway from dorsal aorta to the dorsal longitudinal anastomotic vessel. (B) Effect of cinnamon extract on zebrafish development. (C) Morphology of zebrafish as affected by exposure to cinnamon extract. Exposure time of cinnamon extract was 16 h. Age of zebrafish was 24 hpf when photographed. Treatments were done in triplicate, and experiment was conducted twice. The data represented are pooled from two experiments. Sample size *n* = 30. *P < 0.05; **P < 0.01.

The above hypothesis was also tested in zebrafish as an animal model system. Zebrafish is a vertebrate system and is extensively used for drug screening and as a model for angiogenesis. ISV in zebrafish sprouts from the aorta, runs between each pair of somites, and connects to the dorsal longitudinal anastomotic vessel (Childs et al. 2002). ISVs appear to sprout from the aorta (Fig. 3A), beginning at the 24-somite stage (21 hpf; Fouquet et al. 1997). VEGF is expressed strongly between 18 and 19 hpf in zebrafish embryos. We therefore intended to expose the cinnamon extract at gastrulation stage (8–10 hpf; Liang et al. 2001) to ascertain the inhibitory effect of cinnamon before the onset of VEGF expression in the embryos. We also wanted to investigate if the inhibition of VEGF by cinnamon is reversible upon rescuing the zebrafish embryos from cinnamon exposure. We therefore conducted two sets of experiments in which one group of embryos were exposed to cinnamon from 8 hpf until 24 hpf and later rescued in embryonic media for another 24 hpf and imaged for ISV abnormalities. In another experiment, the embryos were continuously exposed to cinnamon extract between 8 hpf until 48 hpf.

**Figure 4:**
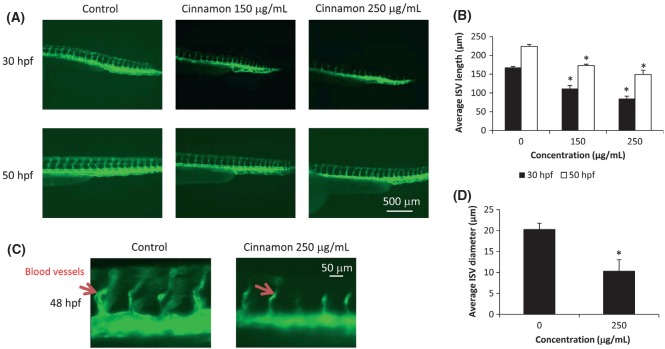
(A) Effects of cinnamon extract exposure on zebrafish intersegmental vessels (ISVs). Zebrafish embryos were incubated in cinnamon extract for 16 h followed by incubation in fresh media stock for additional 24 h. (B) Summary of ISV length in 30 hpf (black bars) and 50 hpf (white bars) embryos exposed to DMSO (0 μg/mL), cinnamon extract at 150 and 250 μg/mL. (C) Zebrafish ISVs at 48 hpf imaged at 4× magnification. (D) Summary of ISV diameter in 48 hpf (black bars) embryos exposed to DMSO (0 μg/mL) and cinnamon extract at 250 μg/mL. GFP fluorescent signal of the ISVs of the transgenic zebrafish embryos was analyzed by fluorescent microscopy. ISVs are indicated by the red arrow. *P < 0.05.

Cinnamon extract exhibited morphological effect in zebrafish at concentrations >250 μg/mL (Fig. 3B). The mortality rate of zebrafish was high at 500 and 1000 μg/mL (60% and 100%, respectively), as shown in Figure 3C. While the effect of cinnamon on angiogenesis has previously been established *in vitro* by Lu et al. (2010), our study aimed to evaluate the molecular mechanism of the antiangiogenic effect of cinnamon using an *in vivo* zebrafish system and HUVEC cell culture model. Data from this study suggest that cinnamon partially inhibited angiogenesis when used at a concentration in the range of 150–250 μg/mL for 16 h (Fig. 4A). The length of the embryo's ISVs at 30 hpf in control group was 167.92 ± 2.42 μm, while the cinnamon-exposed embryos showed significantly shorter ISV lengths of 111.94 ± 7.98 and 85.01 ± 6.69 μm at 150 and 250 μg/mL dosages, respectively (Fig. 4B). The trend was similar even at 50-hpf stage, where the control group embryo's ISV were 224 ± 5.21 while the cinnamon-exposed embryos ISVs were 172.83 ± 3.62 and 149.06 ± 11.73 μm at 150 and 250 μg/mL dosages, respectively. Zebrafish ISVs exposed to 250 μg/mL cinnamon extract were attenuated and underdeveloped as compared with the DMSO-treated control, as shown in Figure 4C. The average diameters of cinnamon-treated embryos were 10.41 ± 2.64 μm compared with the control embryos (20.34 ± 1.42 μm) as shown in Figure 4D.

**Figure 5:**
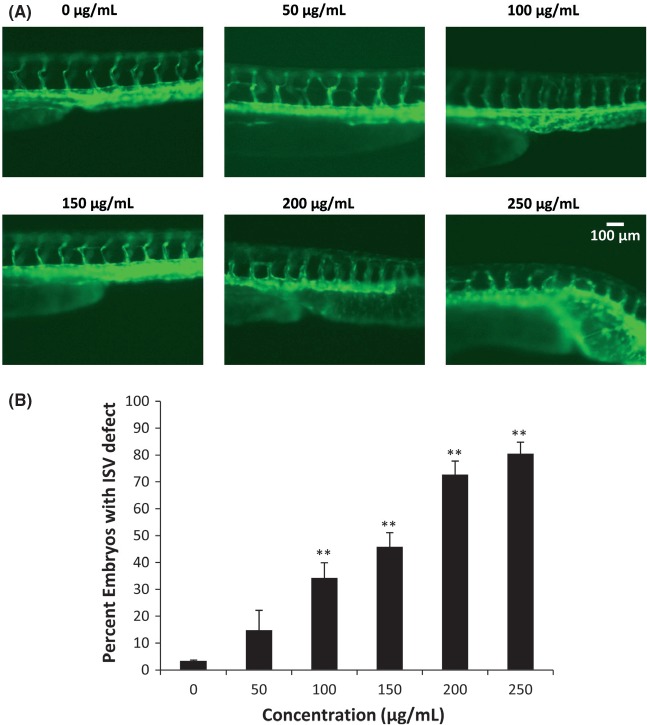
(A) Cinnamon inhibits zebrafish intersegmental vessel (ISV) development accompanied by gross morphological changes in the ventral tail region when exposed for 36 h. ISVs of *TG*(*flk1:GFP*) transgenic zebrafish embryos were visualized by fluorescent microscopy. Zebrafish were 48 hpf when photographed (*n* = 30). (B) Percent of embryos with ISV defect in each treatment (*n* = 30). **P < 0.01.

When exposed to cinnamon extract for longer duration (36 h), embryos showed gross morphological defects accompanied by aberrant initiation of angiogenesis (Fig. 5A). The ventral tail region exhibited stunted growth of the blood vessel, accompanied by loss of the guidance cue of the sprouting angiogenic vessel. We found that cinnamon exposure at 50 μg/mL resulted in only slight impairment of ISV formation. However, at higher concentration led to severe defects in ISV formation in most embryos (Fig. 5B). This effect was more pronounced at higher doses (>200 μg/mL), although severe deformity was also observed. The acute deformity can be attributed to the toxic effect of cinnamon at high concentrations.

## Discussion

This study showed that cinnamon extract inhibits angiogenesis by mediating PKC-dependent activation in HUVEC cells. PKC is a key regulatory enzyme involved in many cellular processes (Nishizuka 1992). PKC is known to be an important factor in the vasculoendothelial system. Activation of PKC by a phorbol ester, such as TPA, induced proliferation (Daviet et al. 1989) and tube formation (Montesano and Orci 1985) of cultured endothelial cells, and caused angiogenesis *in vivo* (Morris et al. 1988). Takahashi et al. (1999) showed that the VEGF receptor tyrosine kinase activated phospholipase-C gamma (PLC-γ) and utilized its enzymatic product diacylglycerol for activation of the PKC/Raf-1/MEK/MAP kinase pathway as the major signaling mechanism for cell proliferation.

Our results strongly suggest that cinnamon extract inhibits TPA-induced PKC activation of MAPK. Additionally, the mRNA expression of VEGFR1 and VEGFR2 were found to be downregulated in HUVEC cells treated with cinnamon extract. The inhibition of VEGFR1, VEGFR2, and PKCα in the presence of cinnamon extract in HUVEC cells stimulated by TPA provides a critical link between the bioactive compounds in cinnamon extract and PKC-mediated activation of angiogenesis. Taken together, the results suggest that cinnamon extract regulates the signal transduction pathway involving activation of TPA-responsive PKC isozymes, especially PKCα, in downregulating VEGF receptor expression in HUVECs.

Previous studies have shown that procyanidines present in cinnamon inhibit angiogenesis in a chick aortic ring assay (Lu et al. 2010), and diminishes tumor growth (Kwon et al. 2010), angiogenesis, and vascularization by inhibiting the levels of proangiogenesis factors, Cox-2, and HIF-1α in tumor tissues (Kwon et al. 2009). Our finding provides novel insights into the role of cinnamon in regulating the mechanism of the endothelial signaling pathway involving PKC-mediated activation of MAPK and angiogenesis using a zebrafish system and a cell culture model.

Cinnamon extract inhibited angiogenesis in rapidly developing blood vessels in the ISVs of zebrafish embryos between 8 and 48 hpf. No significant antiangiogenesis was observed in fully developed ISVs exposed to cinnamon extract (data not shown). The exposure to cinnamon extract during the gastrulation period had a significant effect on the new blood vessel formation in ISVs compared to postgastrulation treatment with the extract. This signifies that the cinnamon extract did not cause any vascular disruption in normal vasculature, but likely inhibited the formation of new blood vessels during angiogenesis.

In conclusion, this study demonstrated that cinnamon extract exhibits antiangiogenic activity on endothelial cells in the zebrafish embryo and HUVEC angiogenesis models. We have shown *in vitro* that cinnamon extract exerts its inhibitory activity in PKC-mediated MAPK activation. The results suggest that MAPK inhibition by cinnamon extract may regulate the VEGF receptor expression. Therefore, the antiangiogenic effect of cinnamon extract on endothelial cells at nontoxic concentrations makes it an excellent candidate for use as a chemopreventive agent against tumors or other chronic inflammatory diseases.
